# Web-Based Experience Sharing Platform on Medical Device Incidents for Clinical Engineers in Hospitals

**DOI:** 10.1007/s40846-018-0441-7

**Published:** 2018-07-27

**Authors:** Mei-Fen Chen, Cheng-Lun Tsai, Yung-Hsin Chen, Yu-Wen Huang, Cheng-Ning Wu, Ching Chou, Chia-Hung Chien, Pei-Weng Tu, Tsair Kao, Kang-Ping Lin

**Affiliations:** 1 Electrical Engineering Department, Chung Yuan Christian University, 200 Chung Pei Road, Chung Li District, Taoyuan City, 32023 Taiwan, ROC; 2 Biomedical Engineering Department, Chung Yuan Christian University, Taoyuan City, Taiwan, ROC; 3 Technology Translation Center for Medical Device, Chung Yuan Christian University, Taoyuan City, Taiwan, ROC; 4 Food and Drug Administration, Ministry of Health and Welfare, Taipei, Taiwan

**Keywords:** Medical device, Clinical engineering, Experience sharing

## Abstract

The aim of this study was to establish a web-based platform for exchanging medical device management and maintenance experiences to enhance the professional competency of clinical engineers (CEs), which ensures the quality of medical devices and increases patients’ satisfaction with medical services. Medical devices play an essential role in diagnosis and disease management. CEs are responsible for providing functional medical devices that contribute worthwhile functions to a medical service to improve patients’ health and safety. The purpose of the platform is to facilitate collection and sharing of medical device incidents experiences to improve CEs’ capability. To provide useful and practical information for CEs, an event review committee, composed of experts with more than 20 years of clinical engineering experience who were recruited as reviewers, was established under the platform. Cases submitted to the platform were required to have comprehensive descriptions of the device and events. Each case was evaluated by at least two reviewers based on five evaluation indices: (1) severity, (2) breadth, (3) frequency, (4) insidiousness, and (5) correctness. After being reviewed, each final report was published on the platform to be shared with the event submitters and other members. The results show that 116 staffs from 32 different hospitals, registered to join this platform. From January 2015 to December 2016, 70 events were submitted with 56 reports. This study also assessed the platform’s benefits for CEs. A total of 93 respondents completed a questionnaire survey: 93% of the CEs agreed that the information from the platform helped them do their job. The web-based platform has high value as an experience-sharing interface for medical devices. The CEs obtained extremely useful information from the platform for medical device management and their daily duties. This study provided an online training model with systematic methods to improve the quality and effectiveness of medical device management.

## Introduction

High-quality medical devices are crucial for diagnosis and disease management. Proper medical service procedures rely on not only the professional knowledge of physicians but also the functional performance of medical devices [1]. A low-quality medical device is a key risk factor for poor medical services and patient safety. Clinical engineers (CEs) and biomedical equipment technicians (BMETs) in a hospital’s clinical engineering department are the main staff responsible for the medical devices [2–4]. Therefore, CEs and BMETs with professional capabilities and experience with medical devices play a critical role in patient safety. The roles of the clinical engineering department are multiple, and two of the core functions are risk management and quality assurance [2, 3]. Experiences on medical device use are the most effective feedback for maintaining device quality and reducing risks to patients; therefore, several postmarket surveillance systems have been launched to collect adverse event and incident data on medical devices. Examples include medical device reporting (MDR) for the United States Food and Drug Administration [5], medical device market surveillance and vigilance for the European Commission [6], and medical device adverse events reporting (ADR) for the Taiwan Food and Drug Administration [7]. CEs maintain various medical devices in hospitals daily and have firsthand information on medical device use, which could be the most valuable source of event data for a postmarket surveillance system. Medical devices are designed based on various technologies and have complex electromechanical and computer-based system components. It is difficult for CEs, especially juniors, to concurrently solve medical device problems and promote quality assurance. Therefore, a collaborative mechanism to access information on the appropriate responses to adverse events is invaluable. Medical devices are distributed to different hospitals; however, the usage and environments in which the same types of medical devices applied are very similar; CEs working for different medical service providers probably face the same adverse events and product problems. If CEs and their peers working in other hospitals could share their practical experiences on medical devices, working effectiveness would be increased and personal professional competency would be improved.

Experience is important in learning and knowledge acquisition. Through each experience, new knowledge and skills can be obtained [8, 9]. In simulation-based medical education, real-life clinical experience is also required to ensure that learning can be implemented into actual practice [10, 11]. Practical experience with medical devices is significant for CEs working in hospitals. However, most junior CEs lack practical experience in areas such as operating a variety of medical devices, managing medical device adverse events, evaluating the risk level and implementing proper action based on risk control. To overcome the problems of insufficient resource access for junior CEs, the effective remedies include learning through experience and querying senior CEs for advice. CEs work with a variety of medical devices and types of technology; thus, there is a demand for practical suggestions that improve medical device usage and opportunities for continuing education. Senior CEs have experiences in medical device quality management and event analysis. If their experiences could be systematically shared, the demands of junior CEs might be met.

This paper presents a web-based platform for sharing the experiences of CEs on medical device incidents, which is expected to improve the exchange of practical information between hospitals. Different experiences with medical device incidents, termed “events” in this paper, were collected on a website. The platform system’s structure and procedure are described in the Methods section. The data of the platform are provided in the Results section. The benefits of this study are summarized and discussed in the Discussion and Conclusions section.

## Methods

**Fig. 1:**
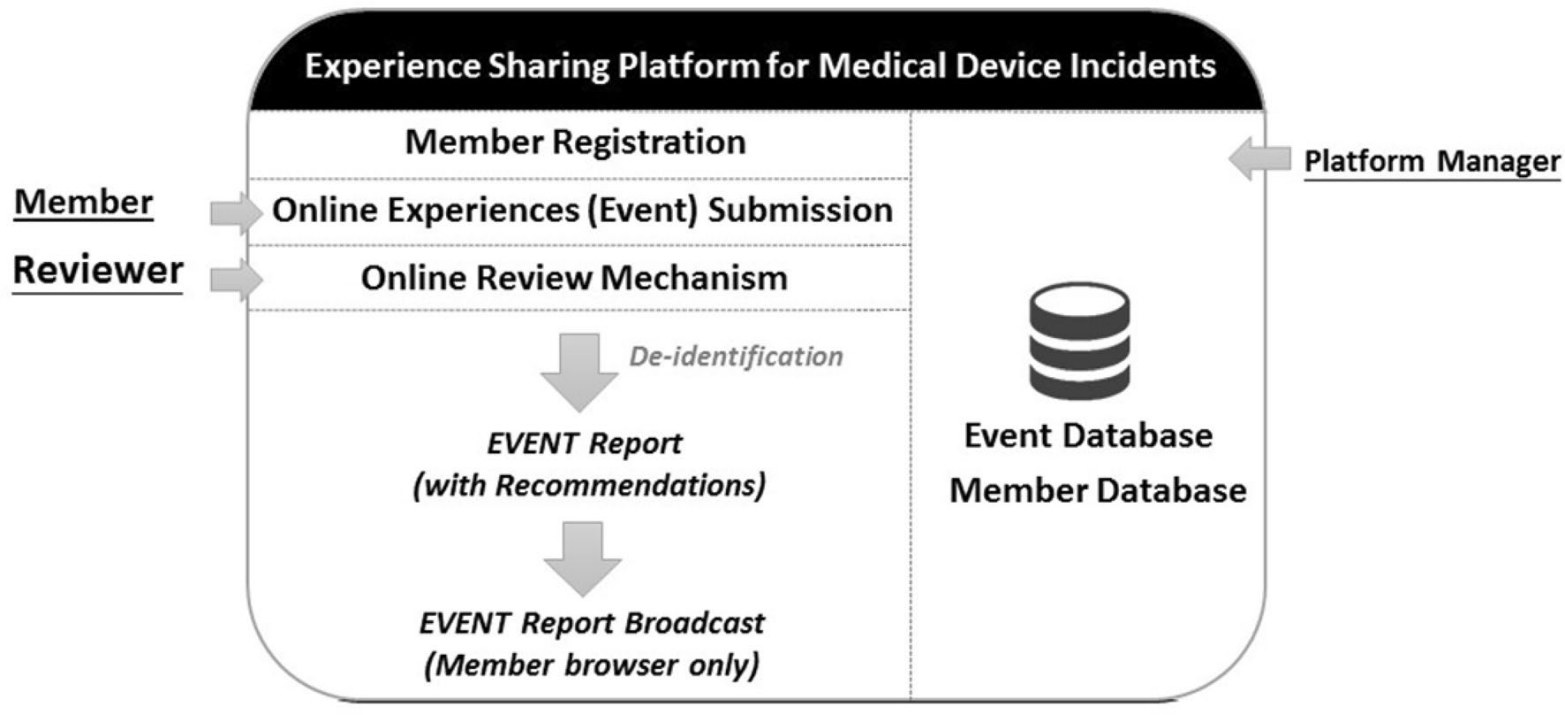
System structure of the platform

The sharing platform for medical device incident experience is described in three parts: registered participants, experiences shared on the platform, and web-based system operations. The platform structure is shown in Fig. 1, including member registration, event submitting, event reviewing, and reporting. A detailed description is provided as follows.

### Participants in the Platform

Participants were classified into three categories as follows:

1. **Member** Any medical institutions could sign an agreement to be a *“seed hospital”* of the platform. Staff with responsibilities related to medical devices in the seed hospital could register as members on the website. CEs, junior CEs, junior biomedical engineers, general affairs personnel, procurement personnel, nurse leaders, and health information engineers were eligible to use the platform. However, CEs and junior CEs were the most welcome.
2. **Event reviewer** Senior CEs and biomedical engineering experts were invited to be the event reviewers. The reviewers were required to have more than 20 years of work experience in clinical engineering, medical device management, systematic integration, medical device regulation, or healthcare technology management in hospitals. The role of reviewers was to provide practical suggestions based on their professional experience to guide members to solve medical device problems.
3. **Platform manager** The platform managers managed the permissions of members and reviewers, maintained daily platform operation, and maintained a database.

### Experiences Shared on the Platform

The experiences shared on the platform focused on difficulties in using medical devices and incidents involving them. Any questions related to the life cycle of the devices in hospitals were welcome. According to the content, events were classified into three types as the follows:

1. **Medical device management problems** Problems related to medical device management, such as new medical device evaluations, service contract reviews, purchase specifications, acceptance, and disposal could be submitted to the platform for suggestions.
2. **Medical device maintenance and troubleshooting** The hospitals’ clinical engineering departments are mainly responsible for the maintenance and troubleshooting of medical devices, where most of the problems are encountered. Members could submit their concerns from regular work to the platform for advice.
3. **Medical device events demonstrated** The events demonstrated on the platform were chosen by reviewers according to their practical usage experience with medical devices, especially the general, common, and important devices in hospitals, such as ventilators, hemodialysis machines, and defibrillators. For this type of event, not only the context but also the troubleshooting of the event need to be provided on the platform.

### System Operation

**Fig. 2:**
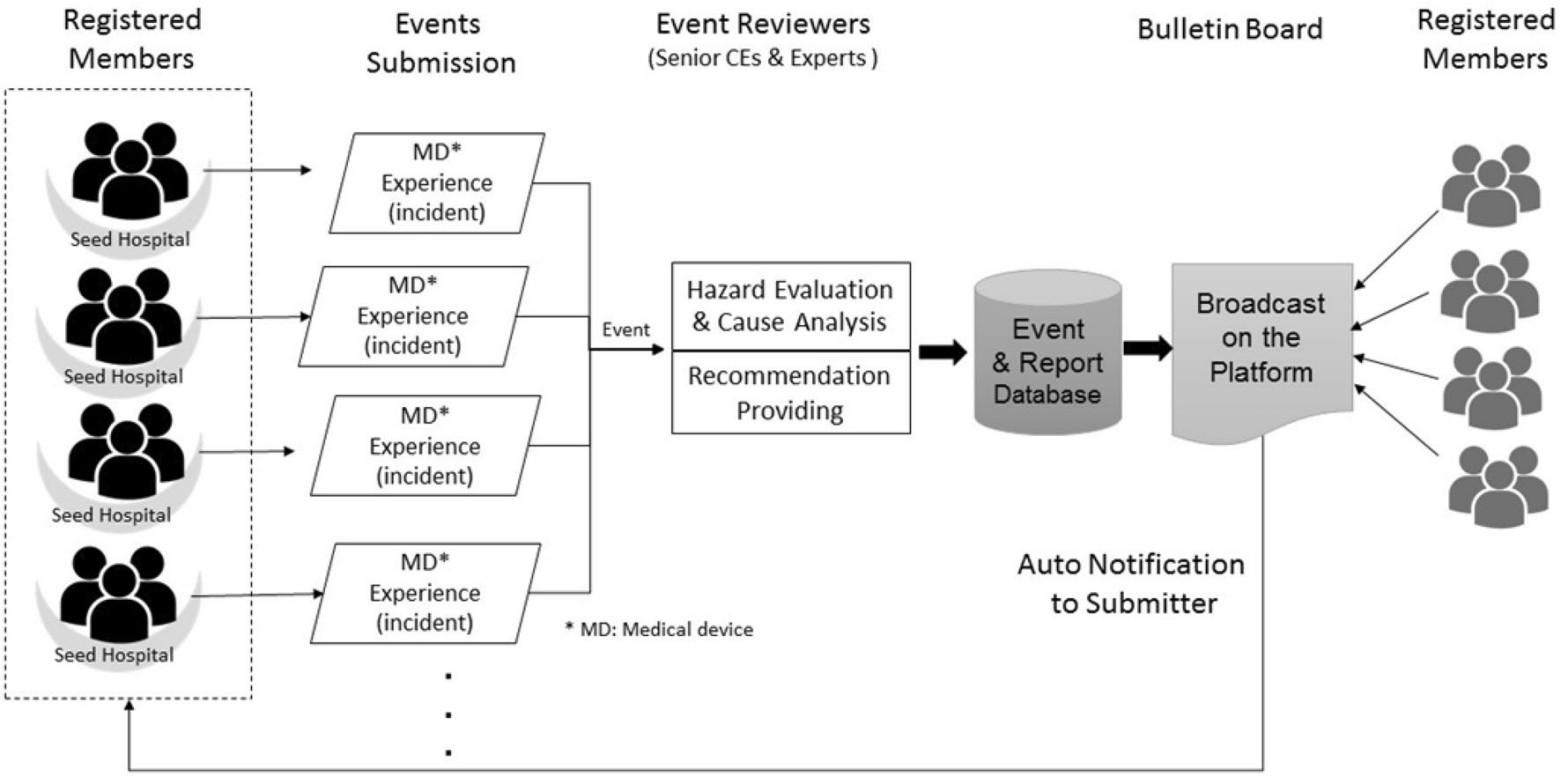
Experiences-sharing process flow of the platform

The Linux operating system with a Microsoft MySQL database and the Hypertext Preprocessor (PHP) programming language were applied in the platform, which can be accessed at the following uniform resource locator (URL): http://md-share.cycu.edu.tw. Based on the aforementioned definitions of participants and experience, an experience-sharing process flow was proposed as presented in Fig. 2. With the approval of the seed hospital, the members provided their experiences of problems with medical devices in practice as the events. Each event was reviewed using the platform’s standard review procedure, and suggestions were provided to the user in the last step. Final reports were combined with the event information and review results, and then posted on the platform.

Establishing an experience-sharing platform is not difficult, and the most important steps are creating an event collection procedure and designing a review mechanism to discern the most useful and practical information. The event collection procedure and review mechanism are described as follows.

#### Event Collection

The members were required to fill in an online form before submitting an event to the platform to ensure that the event information was adequate and consistent. The details required on the form are listed as follows (Table 1): (1) contact information of the submitter, which was deidentified; (2) basic information about the device, including the product’s name, manufacturer, and device license number; (3) event description detailing where, when, how the event occurred and who was involved; and (4) supporting information to help understand the event, including environmental information, photos, and statistics. Additionally, the submitter was encouraged to note any action or plan resulting from the event, such as corrections.

**Table 1:** Data requirements for online event submission

Items	Contents
(1) Contact information of submitter	Submitter’s name and contact phone number Note: Submitter’s name will not be shown in the report and public document
(2) Device basic information	Product name, manufacturer, marker license No., manufacture date, intended user, etc.
(3) Event description	Where and when the events occurred, how event happened and who was involved
(4) Supporting information	Environment information, such as gas, water or electricity supply system, etc. Information to help understand events is welcome, such as device photos, statistics data

#### Online Review Mechanism

**Part 1 Evaluation Indices**

Employing a standard evaluation procedure and consistent data format facilitated the subsequent data analysis and management. The reviewers reached a consensus on event analyses after several meetings and discussions. Each event was analyzed based on five evaluation indices (EIs): (1) severity, (2) breadth, (3) frequency, (4) insidiousness, and (5) correctness [12–15]. Each EI was graded from 1 to 10 (Table 2). Definitions of the five EIs are provided as follows.

**Table 2:** Event evaluation indices

Evaluation indexes	Brief description	Grade
(1) Severity	For severity of an event, concerned on the combination effects of injury and hazard to patient and the environment	1–10
(2) Breadth	Does the event affect great number of people within one facility or multi-facility?	1–10
(3) Frequency	The criteria for grading are to consider the probability of occurrence of the event	1–10
(4) Insidiousness	Does the problem difficult to recognize? Could it lead to downstream errors?	1–10
(5) Correctness	Does the problem affect the data accuracy?	1–10

1. **Severity** Each event received a grade from 1 to 10 points according to its severity. The criteria to define the severity of an event were derived from the ISO 14971 [15] requirements. The severity of the event was classified into five levels: negligible (2 points), minor (4 points), serious (6 points), critical (8 points), and catastrophic (10 points).
2. **Breadth** The criteria to define the breadth of an event were based on the range of its effect. An event that involved one person or a single device received a grade of 1 or 2. An event affecting more than three people or two units received a grade of 5. An event affecting at least 10 people or five units received a grade of 10.
3. **Frequency** The grading criteria were based on the event’s probability of occurring. Both the number of devices and incidents were considered. For example, a hospital has 300 electrical beds and 30 patient monitors, and each type of the devices is involved in 3 incidents during a year. The reviewer may grade 1–3 for the electrical bed and 5–8 for the patient monitor because the incident probability of a patient-monitor event is higher than that of an electrical-bed event. In the review mechanism, an event that seldom occurred was graded 1 or 2. Events that frequently occurred during a certain period of time (which was defined as 1 year in this platform) or were associated with several devices with similar problems received higher grades as empirically decided by reviewers.
4. **Insidiousness** To recognize and evaluate the insidiousness of an event relied on experts having comprehensive experience in operating multiple medical devices. The criteria to grade the event’s insidiousness depended on whether the event was difficult to identify or it led to subsequent process errors. If the event was easy to identify, which means that the problem was easy to identify, it received a low grade (less than 5). If the event might affect the next process and appear in the next stage, it received a grade of 5. If the event was difficult to identify and other related systems might be affected, it received a grade of 10.
5. **Correctness** The criteria to grade the event’s correctness were based on whether the event affected data correctness or completeness for diagnosis and treatment. If the data were not affected, it would receive a grade of 1. If the event could cause missing information or a data transmission delay, but the problems could be corrected using a follow-up process, it received a grade of 5. If the data might not be recovered, it received a grade of 10.

**Fig. 3:**
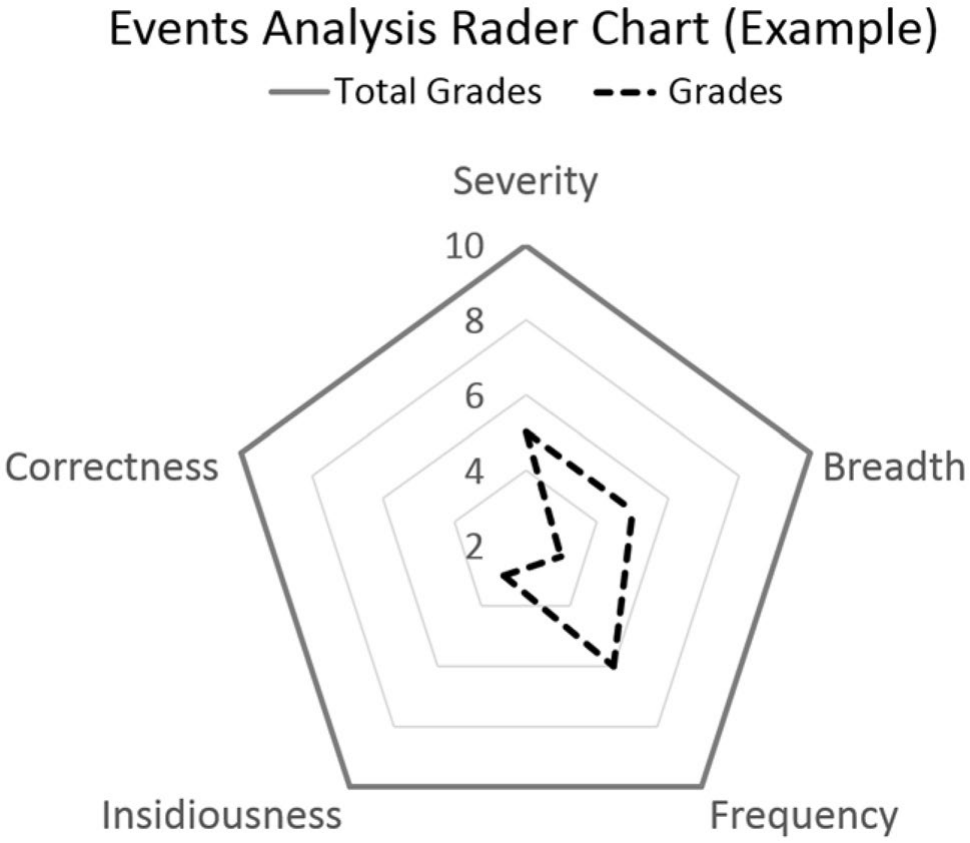
Event hazard analysis demonstrated in radar chart

Each event was demonstrated using a radar chart with the five EIs (Fig. 3). The radar chart is displayed in the report as a reference for the hazard level of an event.

**Part 2 Cause Analysis**

For further analysis, the reviewers evaluated the proportions of causes for each event. Five major causes were identified on the platform based on the quality control analysis [16–18].

1. **User factor** User factors include abuse of the device, accidental misconnection, device misassembly, failure to perform preuse evaluations, improper connection, incorrect control settings, and failure to read the label.
2. **Device factor** Device factors include abuse of the design, labeling error, device failure, device interference, component failure, and packing error.
3. **External factor** External factors include medical gas, vacuum, power, and water supply.
4. **Support system factor** Support system factors include errors in hospital policy, lack of training, and insufficient purchase evaluation.
5. **Environment factor** Environment factors include an unsuitable structure in the hospital building, changing temperature and humidity levels, and improper storage.
6. **Other factor** Factors that cannot be classified into any one of five groups are categorized as “other.”

The total proportion of the six factors was 100%. The reviewers decided the distribution proportion of each factor according to the information provided in the event’s description and the reviewer’s professional specialty.

**Part 3 Recommendations**

In the final part of the evaluation, reviewers wrote a short summary and recommendations for each event, which provided the basis for members to take corrective action. The recommendations were made by each reviewer based on their working experience and integrated knowledge. Additionally, reviewers made suggestions as to whether or not the event should be submitted to the official ARD through the seed hospital’s internal procedures.

**Fig. 4:**
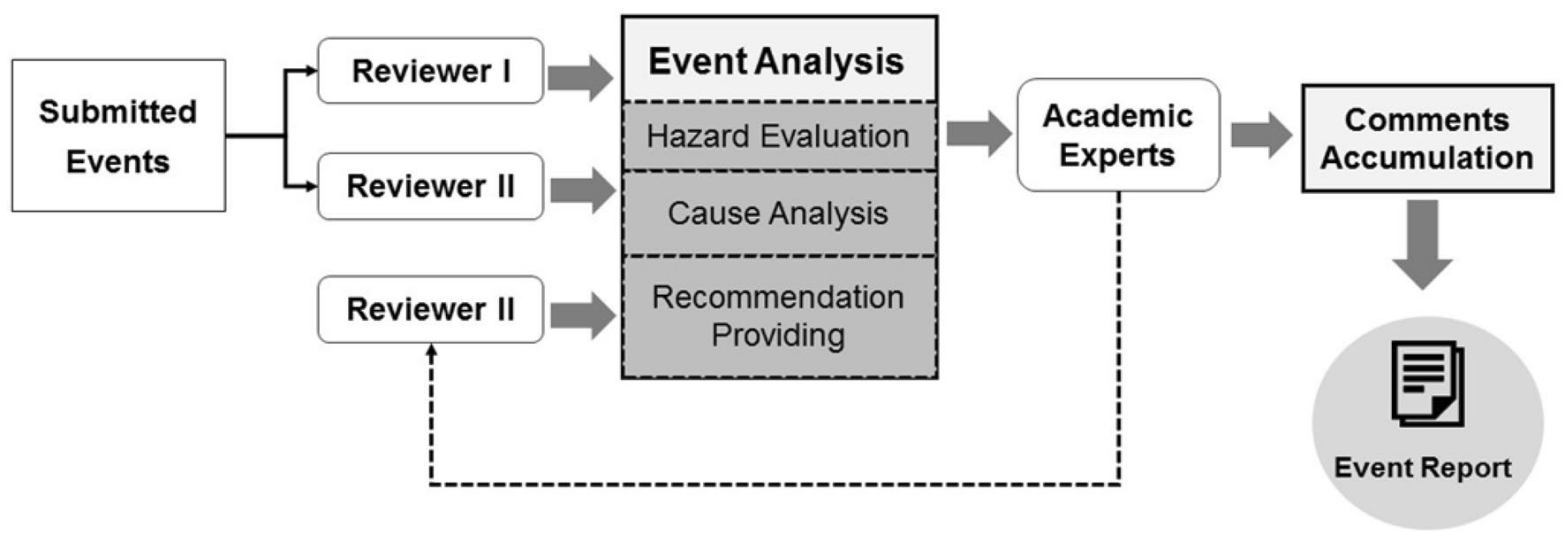
Workflow of online review mechanism

Figure 4 depicts the workflow of the review mechanism. The evaluation procedure, including scoring of the five EIs, was implemented online case by case. The reviewers were required to log into the website to review events, and two reviewers independently reviewed each event to attain objective and practical suggestions. Experts in academia were invited, not as reviewers, to finalize the content and suggestions. The experts had to evaluate whether any inappropriate information was presented in the content. If not, the final report would be completed. If there were conflicts between the EI grades and suggestions, a third reviewer was called on and the report would be finalized with three reviewers’ opinions. Generally, a review process should be completed within 15 days and not longer than 1 month. Each event and its report were collected for the event database and posted on the website for members’ self-learning.

### Questionnaire Survey

This study conducted a performance evaluation to assess the platform’s benefits for CEs.

The participants filled out a three-item questionnaire anonymously.

1. Question 1: How long have you worked in a hospital?
2. Question 2: How often do you browse the website per month?
3. Question 3: How do you think the benefits of the platform influenced your duties?

Feedback collected online during the third quarter of 2016 was used to evaluate the platform’s benefits for its members.

## Results

The results of implementing this platform from January to December 2016 are presented as follows.

### Participants

1. **Members** The platform has approved 116 members from 32 hospitals. Taiwan has 19 medical centers, 81 regional hospitals, and 324 district hospitals. Among them, 5 medical centers, 17 regional hospitals, and 10 district hospitals were approved to be the seed hospitals for the platform. Among the registered members, 35 members were from medical centers, 46 from regional hospitals, and 35 from district hospitals. The numbers of seed hospitals and members are listed in Table 3.

**Table 3:** Numbers of seed hospitals and members registered on the platform

Hospital accreditation level	Total amount of hospital in Taiwan	Seed hospital jointed the platform	Registered members
Medical centers	19	5	35
Regional hospitals	81	17	46
District hospitals	324	10	35
Total	424	32	116
2. **Event reviewers** Eight senior CEs were invited to be reviewers. Each has at least 20 years of working experience and has been a leader of a hospital’s biomedical engineering department. Three experts, distinguished professors of biomedical engineering, collaborated in the event review process to finalize the report.

### Experiences (Events) Collected

A total of 70 events were submitted to the platform in 2016. According to the device risk classification based on the Taiwan Food and Drug Administration regulations [19], 24 events were in Class I, 42 events were in Class II, 2 events were in Class III, and 2 events did not involve a medical device. Only 3% of the events were related to high-risk devices, and the distribution of event numbers with respect to the device risk classification is shown in Table 4. Furthermore, the information listed in Table 5 represents the category distribution for the 70 events, which was based on the Taiwan Food and Drug Administration regulations [20]. Table 5 shows that cardiovascular devices, general hospital and personal use devices, and general and plastic surgery devices are the top three of the 16 categories, and the proportions are 22.9, 21.4, and 15.7%, respectively. None of the 70 events pertained to immunology and microbiology devices, dental devices, neurological devices, or orthopedic devices. Additionally, two reported events, involving an ethylene oxide cylinder and a reverse osmosis water system, were not included in the medical device regulatory category but were still reported by BMETs and were related to a cleaner and sterilizer.

**Table 4:** Distribution of events based on device risk classification

Class^a^	Case number	Proportion (%)
Class I	24	34
Class II	42	60
Class III	2	3
Others	2	3
Total amount	70	100

^a^Device risk classification according to Taiwan Food and Drug Administration regulations

**Table 5:** Distribution of cases according to device category

Code	Category^a^	Device (number)	Event number	Proportion (%)
A	Clinical chemistry and clinical toxicology devices	Blood glucose meter(1)	1	1.4
B	Hematology and pathology devices	Centrifuge(1)	1	1.4
C	Immunology and microbiology devices	–	0	0.0
D	Anesthesiology devices	Resuscitator and accessory(2), anesthesia apparatus(1), ventilator(1)	4	5.7
E	Cardiovascular devices	Patient monitor(4), cardiograph(2), defibrillator(3),electronic sphygmomanometer(1) Vital signs monitor(2), ECMO(1) Cardiovascular patch(1), cardiopulmonary resuscitator(1), warming system(1)	16	22.9
F	Dental devices	–	0	0.0
G	Ear, nose, and throat devices	Audiometer(1), video system(1)	2	2.9
H	Gastroenterology-urology devices	Dialysis machine, video router and accessories	2	2.9
I	General and plastic surgery devices	Surgical Table (5),clip applier(3),electrosurgical generator(2), surgical instrument(1)	11	15.7
J	General hospital and personal use devices	Electric hospital bed(6), Iv pump and accessory(3), catheters(1), cleaner and sterilizer (2), cotton swab(2), vein viewing locator(1)	15	21.4
K	Neurological devices	–	0	0.0
L	Obstetrical and gynecological devices	Fetal monitor(1), fetal monitor(1)	2	2.8
M	Ophthalmic devices	Knives(2)	2	2.9
N	Orthopedic devices	–	0	0.0
O	Physical medicine devices	Frequency therapy unit(2), powered tilt Table (1), warmer(1)	4	5.7
P	Radiology devices	X-ray system(4), ultrasound system(1), PET/CT system(1), MRI(1), accessories (1)	8	11.4
–	Others	Ethylene oxide cylinder(1), reverse osmosis water system(1)	2	2.85
Total			70	

^a^Device category according to Taiwan Food and Drug Administration regulations

The majority of the events caused no damage or harm to patients and medical staff. However, in six events, patients incurred injuries from slight to serious, but not permanent. The injuries included a patient’s skin burning from an electrosurgical unit, a patient falling due to poor quality of a bed component, and low SPO_2_ caused by a ventilator malfunction.

### Events Evaluation Analysis

Among the 70 events, 62 were reviewed online with event reports. Eight events were returned to the submitters for supplemental information. The format of the event report is listed in Table 6. Sixty-two events were evaluated with five EIs, and the total grade distribution for the events is shown in Table 7. To sum up the grade of five EIs for each event, all events received a grade of less than 30, which indicated that the hazard level was not very high, and 71% of the events received a grade between 11 and 20.

**Table 6:** Items and content of event reports

Items	Contents
(1) Device basic information	Product name, manufacture, marker license No., manufacture date, intended user and operator…etc.
(2) Result of cause analysis	Default option list of six factors, including User factor; device factor; external factors; support system factors; environment factor, other factors
(3) Event description	Where and when of the events, how event happened and who was involved in the event;
(4) Representative illustrations	Any picture or drawing uploaded by submitter, the first one will be chosen be the representative illustration of the event
(5) Hazard evaluation	Each grades of five EIs, also shown with Rader Chart
(6) Summary and recommendation	Short summary and recommendation provided by reviewers

**Table 7:** Distribution of events according to the sum of the five indices

Total amount of EIs	Events number	Proportion (%)
1–10	5	8
11–20	44	71
21–30	13	21
31–40	0	–
41–50	0	–
Total	62	100

Regarding cause analysis, many of the events that occurred were caused by more than a single factor, and accidents usually occurred not only because of equipment failure but also human error. Table 8 shows the number of factors assessed by reviewers with proportion over 30%. Table 8 also shows that 53 times are related to device failure, including poor components, packing errors, and inappropriate software settings. In addition, 13 times and 12 times occurred because of support system failures and user factors, respectively.

**Table 8:** Distribution of factors number according to cause analysis

Cause analysis	Number
User factor	12
Device factor	53
Support system failures	13
Processing method failures	6
Environment factor	1
External factors	0

Table 9 presents the number of events assessed according the failure code defined by Gonnelli et al. [21] and Wang et al. [22]. Table 9 shows the incidents of medical devices, which were dominated by *ACC* (accessory and supplies failure) and *USE* (failure induced by use). *UPF* (Unpreventable failure), *PPF* (Predictable and Preventable failure) and *PF* (Potential failure) followed sequentially. The results can provide a supporting reference for device maintenance.

**Table 9:** Distribution of events according to failure code

Failure code^a^	Description	Events number
*NPF*	No problem found	0
*BATT*	Battery failure	2
*ACC*	Accessory failure (including supplies)	18
*NET*	Failure related to network	1
*USE*	Failure induced by use (i.e. abuse, accident, environment conditions)	18
*UPF*	Unpreventable failure, caused by normal wear and tear	7
*PPF*	Predictable and preventable failure	6
*SIF*	Induced by service (i.e. caused by a technical intervention not properly completed or premature failure of a part just replaced)	2
*EF*	Evident failure (i.e. evident to user but not reported)	0
*PF*	Potential failure (i.e. in process of occurring)	8
	Total	62

^a^Failure code: defined in Gonnelli et al. [21] and Wang et al. [22]

In the final part of the reviews for the 62 events, reviewers provided practical suggestions regarding how to deals with these incidents. Reviewers also suggested that 20 events of the 62 should be reported to the official ADR system through the seed hospital’s internal adverse event report procedures.

### Questionnaire Survey

A simple, anonymous, three-question questionnaire survey was conducted, and 93 participants replied. The results are listed as follows (Table 10).

**Table 10:** Anonymous questionnaire survey results

Items								
(1) How long have you worked in a hospital?			(2) How often do you browse the platform?			(3) How do you think of the benefit to your work from the platform information?		
Option	Response	%	Option	Response	%	Option	Response	%
1–3 years	26	28	1–2 times	43	46	Extremely helpful	15	16
4–6 years	30	32	3–5 times	33	36	Very helpful	48	52
7–9 years	22	24	Over 5 times	17	18	Some help	24	26
Over 10 years	15	16				No help	6	6
Total	93	100	Total	93	100	Total	93	100

1. Question 1: How long have you worked in a hospital?
  Of the respondents, 28% worked for 1–3 years, 32% worked for 4–6 years, 24% worked for 7–9 years, and 16% worked for more than 10 years.
2. Question 2: How often do you browse the platform in a month?
  Of the respondents, 46% reported 1–2 times, 36% reported 3–4 times, and 18% reported more than 4 times.
3. Question 3: How do you think the benefits from the platform influenced your duties?
  Of the respondents, 16% believed it to be extremely helpful, 52% believed it to be very helpful, 26% believed it to be helpful, and 6% believed it to be no help.

## Discussion and Conclusions

The platform collected the events related to various medical devices in practice through a case-by-case evaluation mechanism, and practical suggestions were provided to improve medical device management. This benefits not only CEs but also all the involved medical staff. According to the reviewers’ evaluation, the website mechanism provides analysis and suggestions with reports posted on the website, so members can apply this information to their daily hospital duties. The events collected by this platform represent information on practical medical device usage. This not only helps CEs to develop professional competency, particularly junior CEs, but also forms a basis for how quality management systems for medical devices in hospitals could be improved.

After this platform was implemented for 1 year, 70 events were submitted by 116 members, distributed among 32 seed hospitals. Sixty-two event reports passed the rigorous evaluation procedure, and only registered members were allowed to read the online content. According to the results of the questionnaire survey, 94% of the participants agreed that the information from the platform was helpful for their work in hospitals.

Today, obtaining information online is easy, and there are many channels for sharing experience; however, most of the information is not supported by solid evidence. The platform in this study has a rigorous event review mechanism: each event was reviewed by at least two experts to provide neutral, professional, and useful recommendations for the members, and a third expert was required only if there were conflicting views. This is valuable in current experience communication sites, especially for medical device management.

According to the World Health Organization’s policies and a statement from the International Federation for Medical and Biological Engineering regarding the World Health Organization’s reform [23, 24], human resources are critical to the global strategy for health, and professional competence is the key factor to perform healthcare services. By improving staff performance through this platform, internal medical device management and risk control in hospitals can be enhanced; moreover, medical device quality assurance systems can be improved, thus creating a safer environment in hospitals for staff and patients. CEs could use the platform to propose improvements and procedural modifications after adverse events when causes are identified. The establishment of this platform has received a positive response from the participants. Despite the design of the platform requiring optimization, it can be a model for training on medical device quality management in the future.
